# Parameters Affecting the Retention Force of CAD/CAM Telescopic Crowns: A Focused Review of In Vitro Studies

**DOI:** 10.3390/jcm10194429

**Published:** 2021-09-27

**Authors:** Abdullah Kamel, Amr Badr, Gehan Fekry, James Tsoi

**Affiliations:** 1 Department of Prosthetic Dentistry, Faculty of Dentistry, Minia University, Minia 61511, Egypt; dr_amrbadr@mu.edu.eg (A.B.); gogofekry_2010@yahoo.com (G.F.); 2 Dental Materials Science, Discipline of Applied Oral Sciences and Community Dental Care, Faculty of Dentistry, The University of Hong Kong, Hong Kong, China; jkhtsoi@hku.hk

**Keywords:** retention force, double crowns, telescopic crowns, CAD/CAM

## Abstract

Telescopic systems constructed using computer aided design and computer aided manufacture (CAD/CAM) can overcome many drawbacks associated with conventionally constructed ones. Since retention is considered the most important function of these retainers, this scoping review aimed to discuss and summarize the parameters that affect this function in CAD/CAM-manufactured telescopic crowns and to compare their retention force values with the recommended retention force. An electronic search was done in Pubmed and Google Scholar databases using different keyword combinations to find the related articles. Seventeen articles that follow the eligibility criteria for this review were selected and analyzed for detection of each of the tested parameters and their effect on retention force. The parameters tested in these articles were divided into parameters related to design, manufacturing, material type, and test condition. Regardless of the effect of these parameters, the retention force values recorded in most of the selected studies laid within or were higher than the recommended retention force (2.5–10 N), which indicated the need to design and set the combination of materials of telescopic systems according to oral biomechanics.

## 1. Introduction

A telescopic denture is defined as “an overdenture which is a dental prosthesis that covers and is partially supported by natural teeth, natural tooth roots, and/or dental implants” [1]. This type of prosthesis uses the double crown system as retainers or attachments. Such retainers are made up of two crowns; primary crown (inner crown), attached to tooth or implant, and secondary crown (outer crown) attached to the denture [2,3]. These retainers are quite commonly used, at least in Germany and Sweden [4], because of their merits of incorporating proper distribution of force on the abutment, allowing for effective oral hygiene with the maintenance of periodontal health, achievement of good esthetics, and good survival rate [5,6,7,8].

The retention mechanism of the double crown system differs according to its design. In the cylindrical design, the retention is obtained by friction between the parallel walled primary and the secondary crowns [3,6,9,10]. Although this design provides sufficient retention at the beginning [11], it has many drawbacks such as technique sensitivity as it requires precise manufacturing procedures to allow fit between the secondary and primary crown [12]. Moreover, there is a rapid loss of retention due to continuous contact of double crowns which results in progressive wear and excessive force on the supporting structures, so it is only used with strongly supported abutments [9]. In the conical design, the retention is obtained by wedging action. Such design is commonly used because it is less technique sensitive and provides a less damaging force to the supporting abutments due to absence of continuous contact between primary and secondary crowns which will act as a force arm [3,9,12,13]. On the other hand, resilient telescopic design which includes 30-micron spacer between the primary and secondary coping which provides adequate fitting, allows for minimizing the friction between double crowns, and compensates for the mucosal resiliency during functional loading. Such design reduces the possibility of transmitting extra force to the supporting structure, particularly when dental implants are used [14].

Regarding the construction methods of telescopic retainers, conventionally casting technique and electroforming are used. When using the conventional casting method, commonly a lost-wax technique was applied on gold alloy. Nonetheless, due to the high cost, the gold alloy was replaced by non-precious alloys which also provide successful results [15]. The construction procedure of the primary crowns is as follows: (A) a casting wax is modeled directly on the die and trimmed using wax burs mounted on parallelometer; (B) the wax pattern is invested and casted; (C) the cast pattern is checked for fitting on the die; (D) the required parallelism or degree of taper is verified using metal burs mounted on the parallelometer and finally; and (D) finishing and polishing. Regarding the construction of the secondary crowns, casting wax was modeled directly on the primary crowns then invested, casted, finished, and polished [15,16,17,18]. The casting technique needs multiple steps which is considered technique sensitive and requires a highly skilled dental technician to establish accurate fitting with proper retentive force [19,20]. On the other hand, the electroforming method provides adequate retention forces and a good adaptation of the double crowns but is usually limited for construction of secondary crowns. The galvanic process includes deposition of gold on primary crowns or specially prepared die and then the thin formed gold layer was supported by a tertiary structure. The high cost and need for additional equipment are considered as disadvantages.

Recently, the CAD/CAM technique has been adopted in fabricating telescopic dentures, overcoming most of these drawbacks since it is easy to produce a stable retentive force by controlling different design features according to each condition, reduces working time, and minimizes technical errors, and sensitivity [19,21]. Besides, it allows for easy modification of the design before manufacturing [22,23,24,25] and the possibility of constructing primary crowns from a wide range of materials, including tooth-colored materials—such as zirconia and PEEK—which are more esthetic and provide less hypersensitivity and thermal irritation to vital teeth when compared with metal alloys [26,27,28]. Using CAD/CAM can even design all the components altogether including the implant, retainer, and the milled denture at the same time. Thus, using CAD/CAM is particularly useful and advantageous in implant-supported telescopic denture [22].

The dentures should be retentive to provide proper function, the magnitude of retention force should be reasonable to avoid damage to the underlying teeth or implants. This magnitude of the force was mentioned in the literature as ranges that differ according to each situation.

For instance, Pigozzo et al. reported that satisfactory retention force to stabilize the denture lies within the range (5–7 N) [29]. While Stančić and Jelenković reported that the safe range was (5–9 N) to provide denture functionality and avoid damage to the supporting structure [30]. While another study recommended that the minimum accepted force was 2.5 to 3 N and the maximum retentive force per abutment should not exceed 6.5 N [31]. Korber concluded that the retentive force per abutment should be within the range (5–10 N) [32]. With reference to these articles, the range of retentive force from 2.5 to 10 N seems acceptable and was used as the recommended retention force in this review. 

The purpose of this review was to compare the published studies investigating the different parameters that affect the retention force values of CAD/CAM constructed telescopic systems. The retention force values of telescopic crowns tested in these studies with the aforementioned “recommended retention force” would also be compared. 

## 2. Materials and Methods

### 2.1. PICO Question

The PICO question when searching for in vitro studies were as follows:

(P) Population: Models representing a tooth or implant

(I) Intervention: Parameters affecting the retention force results

(C) Comparison: -

(O) Outcome: Retention force

### 2.2. Search Strategy

An electronic search was performed in two major electronic databases: PubMed and Google Scholar, using these key words in combination: retention force, retentive force, retention load, double crowns, telescopic crowns, telescope crowns, and CAD/CAM. The electronic search was completed in June 2020.

### 2.3. Inclusion Criteria and an Exclusion Criteria

The inclusion criteria included in vitro study, studies measuring retention force, published in English, specimen constructed using CAD/CAM technique, studies that tested multiple manufacturing techniques including at least one test group manufactured by CAD/CAM, single-unit testing studies, telescope crowns or double crowns were considered as specimens. The exclusion criteria included studies that were published in any language other than English, studies that measure the retention force of the whole prosthesis.

## 3. Results

**Figure 1:**
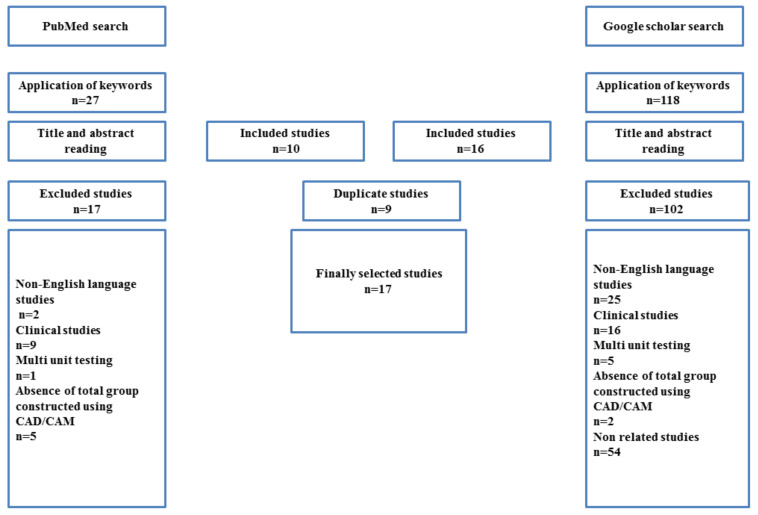
Flow diagram for the search strategy and selection process for the included studies.

The study selection is described in the flow chart in Figure 1. From this search, up until June 2020, 118 articles were found from Google Scholar and 27 from PubMed. After exclusion of duplicates and application of eligibility criteria 17 articles were selected. Table 1 shows the materials, CAD/CAM systems, designs, manufacturing, and testing parameters of the selected studies. Table 2 shows the values of these parameters, specimen’s designs, and their effect on retention force. The CAD/CAM materials of telescopic crowns used in these studies included metals (titanium, titanium alloy, and cobalt-chromium) and non-metals (zirconia, PEEK, and PEKK). The most commonly tested design parameters were taper degree and space setting between the primary and secondary crowns. The parameters related to manufacturing were the usage frequency of milling bur, position during milling and sintering, and surface roughness. Most of the studies used a wet condition with applying a 50 N load. The retention force of zirconia secondary copings was near to recommended retention force when using a 4° taper with 50 N occlusal load and also when using taper of 2° with 25 N occlusal load. Titanium at a 6° taper also provided retention force results within the recommended retention force range. The amount of retention force at different space settings was greatly variable with different materials and testing conditions. The effect of the number of cycles of insertion and removal varied between these studies.

## 4. Discussion

### 4.1. Parameters Related to Computer Aided Design (CAD)

#### 4.1.1. Taper Degree

Thirteen articles tested the taper degree effect on the retention force. Eight of these studies [17,19,21,25,33,34,39,42] in which specimens with 0°, 1°, 2°, 4°, 5°, and 6° taper angle were tested and the results showed that increasing the taper resulted in a decrease in the retention force. On the other hand, three studies [37,38,40], in which specimens with 0°, 1°, and 2° taper were used and reported that the milled group with 2° degrees taper provided more retention than 1° and 0°. The same result was recorded with one of the tested study group done by Merk et al. [35] but in another group, the retention is highest in 1° and lowest in 2°. Another study [36] found that highest retention force was recorded with 2° taper group and lowest recorded with 1° taper.

The factor that should be considered during selection of the taper of the primary crown, according to classical Newton’s second law of motion, can be related to the coefficient of static friction (μ_s_) [33,44], such that

F = μ_s_·N (1)

where F is the retentive force (i.e., maximum frictional force between two surfaces before sliding begins) and N is the force normal to the interface between the two crowns, which is related to the occlusal force L and taper angle θ

N = L/sin θ(2)

Therefore, putting (2) into (1),

F = μ_s_·L/sin θ

Ideally, from 0–90°, a smaller θ or a larger μ_s_ would have a larger value of F. Furthermore, as an example, the coefficient of static friction of zirconia was 0.1, and this value is 0.3 to 0.4 smaller than metal [21] which means that zirconia should have no tolerance on the taper to obtain comparable results with metals. Other studies showed that 5° and 6° degrees can be used and record a retentive force within or above the range of the recommended retention force in titanium and titanium alloy [17,24,33], but zero or near to zero when used with zirconia [21,39]. The same results were obtained in a numerical study [45] which reported that the retention force was zero when a 6° taper used with zirconia. Generally speaking, the μ_s_ of metal under dry condition is higher than ceramic, whereby that for plastic is the lowest if the opposite surface is the same.

When comparing the effect of taper using different manufacturing techniques, one study [17], in which 4° and 6° of taper were tested, the effect of taper was found similar among cast gold alloy, cast Co-Cr alloy, and CAD/CAM constructed titanium groups where 4° taper showed a higher retention force than 6° taper. Another study [34] comparing 0°, 1°, and 2° of taper showed an insignificant difference of varying the taper in cast Co-Cr group and CAD/CAM Co-Cr group. On the other hand, compared to CAD/CAM and electroforming techniques, one study [17] in which tapers of 4° and 6° were used showed that the effect of taper was similar in the electroformed group and CAD/CAM titanium group. For other three studies [34,35,37] in which taper of 0°, 1°, and 2° were used, the effect of taper differed in electroformed group and CAD/CAM zirconia and Co-Cr groups. When comparing CAD/CAM to pressing from pellets and granules techniques, three studies [36,38,40] were found in which taper of 0°, 1°, and 2° were used. Two studies [36,40] showed difference on the taper effect between pressed and CAD/CAM PEEK group, whilst the other study [38] showed these two groups are similar. From these findings, it seems that the manufacturing techniques had a greater effect on the retention force for 0°, 1°, and 2° taper but not for 4° and 6° taper.

Regarding the values of the retention force recorded with each taper, five studies [17,21,24,33,39] tested specimens with 6° using two materials zirconia and titanium. The retention force recorded from the zirconia specimens in two studies [21,39] (0–1.4 N) lied below the recommended retention force. Regarding to the three that tested titanium specimens, retention force values at 50 N load ranged from (6.3–10 N) which lies within the recommended retention force value, while at 100 N load, the retention force range was 13–24 N which lies above the recommended value [17,24,33]. In fact, according to Equation (2), higher occlusal load would induce a higher retention force. Therefore, this is forecastable. In taper 5°, only one study [33] used titanium specimens and the initial retention force values were 35 N which was higher than the recommended retention force value.

In taper 4°, six studies tested zirconia and titanium specimens, while four studies [19,21,39,42] showed that zirconia achieved values which lied within the recommended retention force (8–9.4 N) at 50 N load, while at 100 N load the recorded values were higher than the recommended retention force (15–17.7 N). Again, Equation (2) upholds. Regarding titanium specimens which were tested in two studies [17,33], the initial retention force was 20.8 N at 50 N load and 45.5 at 100 N load, both values considered higher than the recommended retention force. This can be attributed to the larger static friction coefficient between metal to metal than zirconia to metal.

In taper 2°, studies where tested specimens were constructed from different materials combinations including zirconia, PEEK, and Co-Cr, the retention force range of these studies was 14–47 N in different conditions of 50 and 100 N load which is higher than “The recommended retention force”. Six studies [34,35,36,37,40,41] used specimens with taper 1° and the retention force average of PEEK secondary crown specimens was 2.59–10.6 N, which is considered nearly within “the recommended retention force”. However, zirconia and Co-Cr specimens recorded 17.92–26.44 N range which was higher than “The recommended retention force”. Five studies [34,35,36,37,40] used specimens with taper 0° PEEK specimens recorded the retention force range of 3.6 to 13.83 N which was near to “the recommended retention force”, while zirconia and Co-Cr recorded range of 15 to 28.46 N that was higher than “the recommended retention force”.

From the aforementioned, it seems to the taper angle is sensitive to the material used, such that recommended retention force range can be obtained when using titanium at 6°, zirconia at 4° and PEEK at 0° and 1°. A previous study [25] supports part of this conclusion as it reported that the theoretically optimum convergence angle for zirconia would be approximately 3.4°. In addition, although increasing the taper result in a decrease in the retention, the presence of a taper of 1° or 2° is necessary to obtain sufficient retention force. The retention of cones double crowns obtained by wedging effect in which the occlusal force allows for settling of secondary crowns into primary crowns. This result in an elastic strain in the outer crown, especially in the presence of a gap between the double crowns this strain is responsible for providing the retention force [21,24,33,44]. On the other hand, regardless the “recommended retention force”, this can be an indication for the telescope crown to be used in different regions that are biomechanically different. For example, according to Equation (2), if the crown is in posterior molar region that suffer from a higher force, a material with a higher coefficient of friction like metal or a smaller taper angle is necessary in order to withstand the occlusal force. If the telescopic crown is fitted in the anterior region, the material of choice can be extended to ceramic or PEEK that have lower μ_s_ or a larger taper angle.

#### 4.1.2. Space Setting between Primary and Secondary Crowns

During CAD/CAM design, it is necessary to adjust the cement space setting, this setting allows the creation of a gap between primary and secondary crowns. Normally, this gap set at or near 0 μm in the margin area. The gap which begins superior to the marginal area is termed the ‘radial gap’, and the gap which located at the occlusal surfaces is termed the ‘occlusal gap’. The presence of these radial and occlusal gaps is necessary to allow for better adaption of crowns [46]. Nakagawa et al. found a 30-μm space in the occlusal area between primary and secondary crowns when the space setting was 0 μm [39]. Shimakura et al. tested specimens with 0, 50, and 100 μm occlusal gap widths and reported that an increased gap width would increase the retention force [24]. In this study [42], the retention force at 50 N loading was 6.3, 9, 9 N at respective 0, 50, 100 μm occlusal gap width at 4 mm height primary crowns, the retention force at different occlusal gap width are considered within “the recommended retention force”, and 7.8, 15, 16 N at 0, 50, 100 μm occlusal gap width at 6 mm height primary crown. Therefore, the retention force at 0 μm was considered within “the recommended retention force”, while in case of 50 and 100 μm, the retention values were above “the recommended retention force”. Schwindling et al. found that desirable retention was obtained when the occlusal gap and the radial gap were set at 50 μm and 30 μm, respectively, and explained that the presence of this gap compensates for the deviation from the designed geometry after milling and sintering of zirconia [25].

One study [21] tested specimens with 0, 10μm and another one [39] tested specimen with 0, 10, 20 μm gap between primary and secondary crowns and found that no significant difference was found. The significant effect was the taper degree, for example in the first study, with 50 N load application, the retention force was 23 N and 22 N at taper 2°, 8 N and 8 N at taper 4°, and 0 N and 0 N at taper 6°, all on 0 and 10 μm gap. In the second study the retention force was 20, 18, and 16 N at 0, 10, and 20 μm. It is noteworthy that two other studies [19,42] selected the use of the 10 μm spacer setting but with varying test conditions (wet or dry), loads, and tapers. Indeed, if the test condition is wet, i.e., lubricants are taken into consideration, the kinetic coefficient of friction (μ_k_) should also be considered because rubbing also occurs. The rubbing is an action describing the friction between two motion objects, whereas the surface chemistry in micron scale between the lubricant and the object should be known in order to calculate the work being transmitted from the solid object to the lubricant and then to another object. Careful attention must be paid toward studies that used wet condition because research on μ_k_ aspect is lacking in dentistry. Therefore, it should be evaluated in further studies.

The presence of the gap affects the retention of the conical telescopic crowns, because when the occlusal load is applied, there is a slight deformation of the secondary crown. Therefore, the presence of such gap allows the slight deformation while the secondary crown remains intact with its retention force. In absence of this gap, the primary and secondary crowns were already in direct contact. When occlusal load is applied, no deformation is allowed to destroy the retention force (friction) between the two crowns by, say, shear or sliding. Shimakura et al. found that this deformation remains constant with the same load when the gap increased from 50 μm to 100 μm. Thus, it is advisable not to exceed this limit [24].

#### 4.1.3. Height of Primary Crown

Shimakura et al. tested two heights of primary crowns and concluded that increasing the height resulted in an increase in retention [24]. Similar results were reported from other studies that used other techniques, such as conventional casting technique and electroforming technique [9,47,48]. Seven studies mentioned the height of primary crowns, five studies [19,21,39,41,42] used 6, 6.5, and 7 mm height, while one study [33] used 3.5 mm height, and another study [24] used combined heights of 4 and 6 mm. in this study [42] the retention force values lied within and higher than “The recommended retention force” at 4 and 6 mm heights, respectively at 50 N loading. Hence, the increase in retention force as the height of the primary crown increases may be attributed to an increase in the surface area of contact between primary and secondary crowns [49].

#### 4.1.4. Chamfer Depth of Primary Crown and the Thickness of the Secondary Crown

Nakagawa et al. designed primary telescopic crowns with three levels of depth of the finish line (0.6, 0.8, and 1.0 mm) and milled secondary telescopic crowns with three different thicknesses (0.4, 0.8, and 1.2 mm), they found that there were no significant effect of both parameters on the retention force [39]. Four studies mentioned the chamfer depth of specimens, whereas the most commonly used chamfer depth of primary crowns was 0.8 mm [19,21,39,42]. Eight studies mentioned the thickness of the secondary crowns, the most commonly used thickness was 0.4 mm [19,21,33,39,42]. In another study [50] which used conventional casting technique to construct specimens from gold-platinum alloy with 0.4, 0.6, and 0.8 mm thickness of secondary crowns, the results showed that the retention decreased as the crown thickness increased. The effect of different chamfer depth and crown thickness related to the strain that occurs in the secondary crown when the occlusal force was applied, this strain tends to be concentrated near the tooth cervix. Therefore, a large chamfer width of primary crown and a thin secondary crown will result in a large surface friction at the area of contact between double crowns, thus increasing the retention force [39,50,51].

### 4.2. Parameters Related to Computer Aided Manufacture (CAM)

#### 4.2.1. Usage Frequency of Milling Bur

Nakagawa et al. tested telescope crowns milled by a new bur and other crowns milled by older ones (previously work for 6 h), they found that there was no significant difference between both groups on the retention force values [39]. It should be noticed that the cutting efficiency of milling burs depends on the diamond particles and cutting grooves on the surfaces of diamond and carbide burs. The frequent use of these burs would decrease the number of diamond particles, and cutting grooves would become dull that incurs an increase of the surface roughness of the burs which can affect the retention force of the surfaces [52,53]. Additionally, the loss of sharpness of the milling bur may influence the technical precision of secondary crowns and this might affect the retention force [39]. It could be that 6 h may not severely wear the surface of the burs in the Nakagawa et al. study. Strict use of the bur according to manufacturer instructions is necessary.

#### 4.2.2. Position during Milling and Sintering

Nakagawa et al. placed and milled telescope crowns from three different locations on the milling disc (center or periphery or in between) and placed the milled crowns into three different locations on the sintering tray (center or periphery or in between). They found that there no significant effect of different locations on the retention force [39]. This said, the milling and sintering processes that normally affect the dimension of the prostheses seem to be successful in that they have no effect on the precision of the dental prosthesis.

#### 4.2.3. Surface Roughness

Sakai et al. [33] tested double crowns constructed from titanium alloy with different combinations of smooth and rough surfaces. They used specimens with taper 2°, 4°, and 6° with smooth primary and secondary crowns, and taper 6° with different surface roughness combinations, i.e., primary and secondary rough surfaces crowns, smooth primary and rough secondary crowns, and rough primary and smooth secondary crowns. The results showed that, from 0 to 100 cycles, the retention force decreased in all groups. Then in further cycles, the retention force increased in the rough surface groups and still unchanged in the smooth surface group. In addition, the retention force of a smooth surface had higher retention values than a specimen of rough surfaces after 1000 cycles of insertion and removal. In this study, both smooth and rough surface crowns recorded higher initial retention force values than the recommended range. Another study with a different manufacturing technique (electroforming) showed that the surface roughness of the primary crowns affects the retention force as smooth surfaces provide better retention because they provide better adaptation of double crowns with minimal gap distance in between [47]. However, these studies have not evaluated the roughness or the surface texture before and after the crowns’ insertion. Thus, the wear of surface, which can cause friction, is unknown. Caution should be taken while reading these results.

### 4.3. Lot Number and Material Type

Nakagawa et al. used zirconia discs with three different lot numbers to construct telescopic crowns and found that there was no impact of using products with a different lot numbers on the retention force [39]. Multiple materials were tested in the selected studies and found to be suitable for the construction of CAD/CAM double crown systems. These materials can be used in a homogenous form in which the inner and outer crowns are constructed from the same material, or in a heterogonous form in which a combination of different materials is used for constructions.

#### 4.3.1. Zirconia

Zirconia is the most commonly used material for CAD/CAM telescopic crown in the selected studies either as primary [15,17,19,21,25,35,36,39,41,42,43] or secondary [19,21,25,35,37,39,42] crown materials. Zirconia primary crowns are characterized by their wear resistance [17,47,54,55], toughness [56], and low surface roughness and energy which resulted in minimal bacterial accumulation [16,57]. Additionally, its tooth-like color provides good esthetics when the patient removes his denture. Zirconia secondary crowns were tested using 10,000 insertion and removal cycles and the results showed that the loss of retentive force was minimal [42]. Another study [25] tested the retention force of zirconia double crowns till 50,000 cycle and the results showed a slight non-significant increase of the retention force after mechanical aging, while none of the specimens were fractured through or after aging and this was due to the proper selection of the crown thickness. The authors explained the slight increase of retention force after aging by increasing the surface roughness of the interacting surfaces which resulted in increasing of the coefficient of static friction which increases the retention force. On the other hand, [42] tested different cycles of insertion and removal using zirconia specimens and reported that increase the number of cycles resulted in a subsequent decrease in the retention force. As mentioned, the surface conditions and combination of both primary and secondary crowns would incur different outcomes. Yet, the current studies did not investigate the effect of wear and surface morphologies. Thus, further studies might warrant a proper explanation for certain conditions.

#### 4.3.2. Titanium

Pure titanium and titanium alloys used for construction of CAD/CAM telescopic crowns are shown to be successful with prolonged retention [17,24,33]. Çelik Güven et al. compared the retention force of different materials groups used for the construction of telescope crowns using different techniques. When the titanium group were compared with another group which used zirconia primary crowns constructed using CAD/CAM and gold alloy secondary crowns constructed using electroforming, the results showed that titanium recorded a higher retention force with taper 4° and taper 6° at the initial retention force and after 5000 cycles [17]. A prefabricated telescopic crown constructed from titanium (primary crown) and gold alloy (secondary crown) was used for retaining implant superstructures with 5–10 N retention force which persisted after more than 5000 cycles of insertion and removals. The prosthesis survival rate was 100% and the implant survival rate was 98.7% after an average five years of usage [58].

#### 4.3.3. Co-Cr Material

Pre-sintered version Co-Cr milling blocks can be used by CAD/CAM technique to produce telescopic crowns [34,35,37,40]. This pre-sintered stage allows for easy milling and then the milled structures were sintered using sintering furnace. The heating cycle protocol supplied by each manufacturer was followed to obtain the final size, mechanical properties, and density [59]. Furthermore, it was reported that the fracture loads of these structures were similar to the conventionally casted structures [60]. Wagner et al. tested specimens constructed from Co-Cr using CAD/CAM technique, higher retention values were found when compared to casted Co-Cr specimens at 0° and 1° taper, even higher than gold alloy specimens constructed using electroforming at 0°, 1°, and 2° [34]. Without the proof from the authors, the milling might induce the milling grooves on the surface that might increase the friction between two surfaces.

#### 4.3.4. PEEK

From the selected studies, PEEK is considered as the second most commonly used material for secondary crowns construction [36,38,40,41,43]. Schubert et al. compared the retention force of PEEK secondary crowns and electroformed secondary crowns (pure gold). The results of PEEK crowns were slightly less than those for electroformed crowns but it provided a stable retention over a prolonged period of artificial aging with artificial saliva, claiming to simulate a clinical usage period of 10 years [41]. On the other hand, another study [43] showed that PEEK secondary crowns recorded higher retention force than electroformed crowns at the initial force and after 900 cycles of fatigue. Three studies [36,38,40] compared the effect of manufacturing techniques of PEEK (Milled, pressed from pellets, pressed from granules) at different taper degrees, the retention force greatly varied with the taper degree and manufacturing techniques. PEEK can be used for the construction of primary crowns, whatever the material used for the secondary crown because of its ductility and low hardness which allow for good adaptation and marginal fit [37,38].

#### 4.3.5. Polyetherketoneketone (PEKK)

It is a PAEK-based polymer characterized by high biocompatibility, excellent mechanical properties, shock-absorbing ability, and can be fabricated using milling and pressing so it has been promoted to be used as a double crown telescopic material [61]. Only one study [15] tested the retention force of telescope crowns constructed from PEKK. In this study, four materials were used for the construction of primary crowns including PEKK, zirconia, non-precious metal, and gold alloy while PEKK was used for the construction of secondary crowns. The results showed that PEKK primary crown group recorded less retention force than the other groups but with different materials combinations, the retention force increased from 0 to 2000 cycles. After that, the retention remained constant until 10,000 cycles of insertion and removal which resembles around 10 years of clinical use. In this study, the initial retention force values with different primary crown materials ranged from 3.6–11 N which considered within “the recommended retention force” but the retention force after 10,000 cycles were (14.8–29.4 N) that considered higher than “the recommended retention force”. Although there is limited literature that clinically tested PEKK for telescopic overdenture, the available studies showed good treatment outcomes and patient satisfaction [62,63]. A study tested the marginal and internal fit of PEKK using a numerical method and reported that it provided excellent results with values within the clinical range [64]. Therefore, testing with wet simulated condition that considers the μ_k_ into account should be read with caution, despite polymeric materials PEKK and PEEK might have a good potential to be the material for telescopic crowns.

### 4.4. Parameters Related to Testing Condition

The loading force and use of dry or wet condition to test the samples can affect the retention force results. Five studies [19,21,24,39,42] tested the effect of press loading with different magnitude of the force reported that retention increased with increasing the applied load. This occurs as more load resulted in more deformation of secondary copings to fit the primary ones. These study retention force values at 50 N are usually nearer to “the recommended retention force” than 100 N. The most commonly used press load was 50 N [17,19,21,24,36,37,39,40,42]. However, the significance of using these loads is not justified. Essentially, when the materials are pressed with a higher load, then the micro-pits on the contacting surfaces between primary and secondary crowns become interlocked micromechanically. Thus, a larger force is needed to separate them. Therefore, there is a need to develop a test that can properly evaluate the retention between the crowns.

Regarding the dry or wet condition of samples, two studies [19,25] tested this effect and found that the retention force increased in presence of wet condition and a further increase in the retention occurred when the amount of applied load increased. This was explained by the fact that when the load was increased, there was a more settling of the secondary crown to the primary one that resulted in minimizing the gap between the double crowns and produced hydraulic adhesion forces. However, adhesion force is not a valid explanation. All liquids are slightly compressible, and under the condition that two crowns are pressed with each other, the liquid appears to ‘lute’ to make a tight air-seal inside the gap. Therefore, no air is trapped between the interfaces and a tight retention is obtained. Several studies [15,17,19,34,35,36,37,40,41] used wet conditions during testing including distilled water, artificial saliva, and saliva substitute with different densities and viscosities that could have different compressibility, i.e., different ability to form the tight seal. Other studies [65,66,67,68,69] which used different manufacturing techniques showed similar results. Nishizaki et al. noted that the retention increased slightly when increasing the viscosity of the artificial saliva [67]. Again, μ_k_ should be further investigated.

### 4.5. Study Limitation

This review is a non-structured study that was not prepared according to PRISMA guidelines of systematic and scoping reviews, this is considered as a limitation of this review as it provides less evidence compared to structured reviews.

## 5. Conclusions

Various parameters affect the retention force of CAD/CAM telescopic crowns, these parameters can be divided into parameters related to design, manufacturing, material type, and testing condition. The tested parameters that produce a significant effect on retention include the presence of an internal and occlusal gap between primary and secondary crowns, increasing the inner crown height, the use of smooth surface crowns, and increasing the loading force in the presence of a wet condition, perhaps due to the μ_k_. The effect of the taper differs according to the material used although increasing the taper results in a decrease in the retention but the presence of a taper of 1° or 2° is necessary to obtain sufficient retention force for the cone crown telescopic system. Some important parameters, such as roughness before and after the insertion and removal of the crowns, have not been evaluated. Parameters were not widely tested and need further studies to verify its effect on retention force such as chamfer depth of primary crown, the thickness of the secondary crown, the usage frequency of milling bur, and the position of specimens during milling and sintering. Regardless of the effect of these parameters, the retention force values recorded in most of the selected studies lied within or were higher than the recommended optimal retention force average (2.5–10 N) indicating the various materials and taper angles in different regions of dentition. Nonetheless, the load is a factor, so a proper test is deemed necessary.

## Figures and Tables

**Table 1:** Data extracted from selected studies.

Author and Year	Materials		Designing and Manufacturing Parameters	CAD/CAM Systems		Testing Condition and Variables
	Inner Crown (IC)	Outer Crown (OC)		Design Software	Milling Machine	
(Shimakura et al., 2008) [24]	Titanium	Titanium	IC height Occlusal gap	-	Dental Cadim system	Load: 50, 100 N Speed: 5 mm/min Dry condition
(Sakai et al., 2011) [33]	Ti alloy	Ti alloy	Taper degree Surface roughness	-	Cincom L 20	Load: 100 N Speed: 50 mm/min Dry condition Fatigue: 1000 cycle
(Wagner et al., 2015) [34]	Co-Cr	Co-Cr Co-Cr (Cast) Gold (Ef)	Taper degree	Ceramill Mind 2.3.0	Ceramill Motion 2	Load: 50 N Speed: 50 mm/min Wet condition
(Merk et al., 2016) [35]	Zirconia Co-Cr	Zirconia Gold (Ef)	Taper degree	Ceramill Mind	Ceramill Motion 2	Load: 5 kg Wet condition
(Merk et al., 2016) [36]	Zirconia	PEEK PEEK (Pressed)	Taper degree	Ceramill Mind	IC: Ceramill Motion 2 OC: Zenotec System	Load: 50 N Speed: 50 mm/min Wet condition
(Schwindling et al., 2016) [25]	Zirconia	Zirconia	Taper degree	Dental Designer	Brain Xpert Pro Cam	Load: 12.5, 25, 50, 75, 100 N Speed: 30 mm/sec Dry/Wet condition Fatigue: 50,000 cycle
(Stock et al., 2016) [37]	PEEK	Zirconia Co-Cr Gold (Ef)	Taper degree	Ceramill Mind 2.3.0	IC: ZENO Tec System OC: Ceramill motion 2	Load: 50 N Speed: 50 mm/min Wet condition
(Stock et al., 2016) [38]	Milled material	PEEK PEEK (Pressed)	Taper degree	Ceramill Mind 2.3.0	ZENO Tec System	Load: 5 kg Speed: 50 mm/min Wet condition
(Çelik Güven et al., 2017) [17]	Titanium Laser sintered metal Zirconia Gold (Cast) Co-Cr (Cast)	Titanium Laser sintered metal Gold (Ef) Gold (Cast) Co-Cr (Cast)	Taper degree	DV3	Exacto 1 EOSINT M 270	Load: 50 N Speed: 10 (Descending), 20 (Ascending) mm/min Wet condition Fatigue: 5000 cycle
(Nakagawa et al., 2017) [21]	Zirconia	Zirconia	Taper degree Cement gap	IC: CATIA OC: Dental System 2015	CAM250i	Load: 50, 100 N Speed: 40 mm/min Dry condition
(Nakagawa et al., 2017) [39]	Zirconia	Zirconia	Taper degree Cement gap IC chamfer width OC thickness Milling bur usage frequency Different lot number Position during milling and sintering	I.C: CATIA software O.C: 3 Shape	CAM 250i	Load: 50, 100 N Speed: 40 mm/min Dry condition
(Wagner et al., 2018) [40]	Co-Cr	PEEK PEEK (Pressed)	Taper degree	Ceramill Mind 2.3.0	IC: Ceramill Motion 2 OC: Zenotec System	Load: 5 kg Speed: 50 mm/min Wet condition
(Kotthaus et al., 2019) [15]	PEKK Zirconia Non precious alloy Gold (Cast)	PEKK	-	Millhouse GmbH	M1 Zirkonzahn	Load: 200 N Speed: 120 mm/min Wet condition Fatigue: 10,000
(Nakajima et al., 2019) [19]	Zirconia	Zirconia	Taper degree	I.C: CATIA V5 OC: Dental System 2015	CAM 250i	Load: 25, 50, 100 N Speed: 40 mm/min, Dry/Wet condition
(Schubert et al., 2019) [41]	Zirconia	PEEK Gold (Ef)	-	Modellier	M1 Zirkonzahn	Load: 20 N Speed: 60 (Descending), 10 (Ascending) mm/sec Wet condition Fatigue: 10,950 cycle
(Yoshikawa et al., 2019) [42]	Zirconia	Zirconia	Taper degree	IC: CATIA V5 OC: 3 shape Dental system 2015	CAM 250i	Load: 50, 100 N Speed: 40 mm/min, Dry condition Fatigue: 10,000 cycle
(Milić-Lemić et al., 2020) [43]	Zirconia	PEEK Gold (Ef)	-	Dental System Premium 2014	Wieland dental CNC	Fatigue: 900 cycle

Co-Cr: cobalt–chromium; Cast: casting; Ef: electroforming.

**Table 2:** Design features of telescope crowns used in each study and retentive force values recorded with each parameter in CAD/CAM groups and other reference groups.

Author and Year	CAD/CAM Materials		Telescope Crowns Design	Taper Degree	Retentive Force Values (N)	Combined and Non CAD/CAM Materials	Retentive Force Values (N)	
	IC	OC						
(Merk et al., 2016) [36]	Zirconia	PEEK		0°	13.83	OC: PEEK (Pressed) Pellets (P) and granules (G)	P: 22.83	G: 15.87
				1°	6.07		21.06	27.00
				2°	14.1		19.84	19.05
(Schwindling et al., 2016) [25]	Zirconia	Zirconia	OC Thickness: 1 mm	1°	L/F: 0.63, 0.041, 0.026 (Dry, ∆ L/F Wet, ∆ L/F 50,000 cycle)	-	-	
				2°	L/F: 0.53, 0.041, 0.026 (Dry, ∆ L/F Wet, ∆ L/F 50,000 cycle)	-	-	
(Nakagawa et al., 2017) [21]	Zirconia	Zirconia	Height of IC: 6.5 mm Chamfer Depth: 0.8 mm OC Thickness: 0.4 mm CG: 0, 10 μm	2°	50 N: 23, 22 (CG: 0, 10 μm) 100 N: 47, 45 (CG: 0, 10 μm)	-	-	
				4°	50 N: 8, 8 (CG: 0, 10 μm) 100 N: 15, 14 (CG: 0, 10 μm)	-	-	
				6°	50 N: 0, 0 (CG: 0, 10 μm) 100 N: 0, 0 (CG: 0, 10 μm)	-	-	
(Nakajima et al., 2019) [19]	Zirconia	Zirconia	Height of IC: 6.5 mm Chamfer depth: 0.8 mm OC Thickness: 0.4 mm CG: 10 μm	2°	25 N: 9.7, 12.2 (Dry, wet) 50 N: 20.1, 22.6 (Dry, wet) 100 N: 43, 46.3 (Dry, wet)	-	-	
				4°	25 N: 4.1, 4.9 (Dry, wet) 50 N: 9.4, 10.5 (Dry, wet) 100 N: 17.7, 19.8 (Dry, wet)	-	-	
(Schubert et al., 2019) [41]	Zirconia	PEEK	Height of IC: 7 mm OC thickness: 0.3 mm	1°	2.59, 2.55 (Initial, 10,950 cycle)	OC: Gold (Ef)	2.86, 3.04 (Initial, 10,950 cycle)	
(Yoshikawa et al., 2019) [42]	Zirconia	Zirconia	Height of IC: 6.5 mm Chamfer Depth: 0.8 mm CG: 10 μm	2°	50 N: 20.8, 18.5 (Initial, 10,000 cycle) 100 N: 40.7, 36.5 (Initial, 10,000 cycle)	-	-	
				4°	50 N: 8.5, 6 (Initial, 10,000 cycle) 100 N: 17.1, 12.7 (Initial, 10,000 cycle)	-	-	
(Milić-Lemić et al., 2020) [43]	Zirconia	PEEK	-	-	9.3, 4.1 (Initial, 900 cycle)	OC: Gold (Ef)	7, 3 (Initial, 10,000 cycle)	
(Stock et al., 2016) [38]	Milled material	PEEK	OC Thickness: 1 mm OG:0.5 mm CG: 0.02 mm (taper 1° and 2°) 0.03 mm (taper 0°)	0°	4.29	OC: PEEK (Pressed) Pellets (P) and granules (G)	P: 14.9	G: 11.64
				1°	21.12		17.46	15.11
				2°	29.06		19.73	17.08
(Shimakura et al., 2008) [24]	Titanium	Titanium	Height of IC: 4, 6 mm OC Thickness: 1 mm OG: 0, 50, 100 μm	6°	Height of IC 4 mm 50 N: 6.3, 9, 9 (OG: 0, 50, 100 μm), 100 N: 13, 15, 17.4 (OG: 0, 50, 100 μm)	-	-	
					Height of IC 6 mm 50 N: 7.8, 15, 16 (OG: 0, 50, 100 μm), 100 N: 23, 35, 35.6 (OG: 0, 50, 100 μm)	-	-	
(Sakai et al., 2011) [33]	Ti alloy	Ti alloy	Height of IC: 3.5 mm OC thickness: 0.4 mm OG: 50 μm	4°	45.5, 30.4 (Initial, 1000 cycle)	-	-	
				5°	35, 23 (Initial, 1000 cycle)	-	-	
				6°	24.2, 19.4 (Initial, 1000 cycle)	-	-	
(Wagner et al., 2015) [34]	Co-Cr	Co-Cr	-	0°	28.46	OC: Co-Cr (Cast)	Co-Cr: 14.1	Gold: 2.87
				1°	18.68	OC: Gold (Ef)	18.33	15.67
				2°	17.4		22.77	6.65
(Wagner et al., 2018) [40]	Co-Cr	PEEK	-	0°	3.6	OC: PEEK (Pressed) Pellets (P) and granules (G)	P: 13.3	G: 17.7
				1°	10.6		7.7	15.5
				2°	16.5		13.4	10.0
(Stock et al., 2016) [37]	PEEK	Zirconia	-	0°	16.9	OC: Gold (Ef)	26.1	
				1°	22.8		9.6	
				2°	38.2		14.8	
		Co-Cr		0°	15			
				1°	21.4			
				2°	31.1			
(Merk et al., 2016) [35]	Zirconia	Zirconia	-	0°	17.63	OC: Gold (Ef)	7.73	
				1°	17.92		14.63	
				2°	22.71		11.35	
	Co-Cr	Zirconia		0°	17.38	OC: Gold (Ef)	10.38	
				1°	26.44		22.4	
				2°	16.86		14.74	
(Çelik Güven et al., 2017) [17]	Titanium	Titanium	-	4°	20.8, 20.7 (Initial, 5000 cycle)	IC and OC: Gold (Cast)	19.27, 32.14 (Initial, 5000 cycle)	
				6°	10.1, 17.6 (Initial, 5000 cycle)		14.33, 23.89 (Initial, 5000 cycle)	
	Laser sintered metal	Laser sintered metal		4°	32.89, 32.65 (Initial, 5000 cycle	IC and OC: Co-Cr (Cast)	16.63, 29.36 (Initial, 5000 cycle)	
				6°	10.07, 21.21 (Initial, 5000 cycle)		8.36, 21.08 (Initial, 5000 cycle)	
	Zirconia			4°	-	OC: Gold (Ef)	11.86, 13.26 (Initial, 5000 cycle)	
				6°	-		5.41, 6.27 (Initial, 5000 cycle)	
(Kotthaus et al., 2019) [15]	PEKK	PEKK	-		4.6, 14.8 (Initial, 10,000 cycle)	-	-	
	Zirconia				11, 29.4 (Initial, 10,000 cycle)	-	-	
						IC: Non precious alloy	8.1, 20.6 (Initial, 10,000 cycle)	
						IC: Gold (Cast)	9, 15.1 (Initial, 10,000 cycle)	
(Nakagawa et al., 2017) [39]	Zirconia	Zirconia		2°, 4°, 6°	35.8, 15.9, 1.4			
			Spacer 0 μm, 10 μm, 20 μm		20, 18, 16			
			Lot number (1), (2), (3)		19, 17, 18			
			OC thickness 0.4, 0.8, 1.2 mm		19, 18, 17			
			Chamfer depth 0.6, 0.8, 1 mm		19, 19, 18			
			Milling position (center), (middle), (periphery)		20, 20, 16			
			Sintering position (center), (middle), (periphery)		20, 19, 18			
			load 50 N, 100 N		15, 25			

IC: inner crown; OC: outer crown; CG: cement gap; OG: occlusal gap; Co-Cr: cobalt chromium; Cast: casting; EF: electroforming; L/F: loosening force/fitting force.
